# Amino acid profile and protein quality in tuber and leaf of *Coccnia abyssinica* (Lam.) (Cogn.) accessions of Ethiopia

**DOI:** 10.1002/fsn3.452

**Published:** 2016-12-23

**Authors:** Yenenesh Ayalew, Nigusse Retta, Gulelat Desse, Ali Mohammed, Adey Mellesse

**Affiliations:** ^1^ Collage of Agriculture & Natural ResourcesDepartment of HorticultureDilla UniversityDillaEthiopia; ^2^ College of Natural SciencesCenter for Food Science and NutritionAddis Ababa UniversityAddis AbabaEthiopia; ^3^ Department of Food Science & TechnologyBotswana collage of AgricultureGaboroneBotswana; ^4^ College of Agriculture and Veterinary MedicineDepartment of Post‐harvest ManagementJimma UniversityJimmaEthiopia; ^5^ Debrezeit Agricultural Research CenterEthiopian Institute of Agricultural Research (EIAR)DebrezeitEthiopia

**Keywords:** accessions, amino acid, *Coccinia abyssinica*, leaf, protein, protein quality, tuber

## Abstract

The protein content and amino acid profile of Anchote (*Coccinia abyssinica*) leaves and tubers were determined from ten different accessions taken from Debre Zeit Agricultural Research Center, Ethiopia. Crude protein content was determined by Kjeldahl method and amino acid profile was analyzed using performic acid oxidation and acid hydrolysis by ninhydrin‐derivatized analysis with amino acid analyzer. Crude protein content of Anchote tuber ranged from 10.70% ± 0.26% to 13.72% ± 0.10%, whereas the crude protein content in leaves were ranged between 30.38 ± 0.01% (“240407‐1”) and 35.42 ± 0.05% (“223109‐1”). Total amino acid content ranged from 45.12 to 62.89 and 67.31 to 75.69 g/100 g protein for tuber and leaf samples, respectively. The mean values of essential, conditionally essential and nonessential amino acids were 37.22 & 36.79%; 28.62 & 24.10%; and 34.16 & 39.11% for tubers and leaves, respectively. Arginine in tubers and glutamic acid in leaves ranked the highest of all amino acids; while the least dominant essential amino acid was methionine in both parts. Among the essential amino acids, leucine was dominant in all accessions tested with values ranged from 3.12 to 5.32 g/100 g protein in tubers and from 5.15 to 5.65 g/100 g protein in leaves. In general, the average amino acid content was higher in the leaves (71.08 g/100 g protein) compared to the tubers (51.11 g/100 g protein). The nutritional quality of *Coccinia abyssinica* leaves and tubers range as follows: total essential amino acids (TEAA)/ total amino acids (TAA) (37.57 & 36.82%), TEAA/total non‐essential amino acids (TNEAA) ratio (0.60 & 0.58), The predicted protein efficiency ratio (P‐PER) (1.22 & 1.80), Essential amino acid index (EAAI) (35.28 & 53.93%), Predicted biological value (P‐BV) (26.76 & 47.09%), Nutritional index (4.11 & 17.71%), and Amino acid score (73 & 108) for tuber and leaf sample, respectively. A significant variability was observed in protein and amino acid profile among accessions and plant parts, and the leaf part were found to be richer in protein content and associated nutritional quality.

## 1 Introduction

Anchote (*Coccinia abyssinica (Lam*.) (Cogn.)) belongs to *Cucurbitaceae* family, one of the most economically important families of plants (Schaefer, Heibl, & Renner, 2009). Among 30 species registered under the genus *Coccinia*, ten species found in Ethiopia of which *C. abyssinica* is cultivated for human consumption (Jeffrey, 1995). *C. abyssinica* (Anchote) is an endemic and potentially valuable crop of Ethiopia principally categorized under root and tuber crops (Holstein, 2012). Its newly growing leaves along with the tendrils are also used as nutritious vegetable served after being cooked (Abera, 1995). The tuber is prepared in different ways for consumption; cooked and served with a fermented spice prepared from coriander (*Coriandrum sativum*), sweet basil (*Ocimum basilium*), ginger (*Zingiber officinale*), garlic (*Allium sativum*) and salt, and also prepare as a soup after drying and grinding into powder (Habtamu & Kelbessa, 1997). It is also cooked for special occasions and holydays in sliced form and pounded after mixing with plenty of butter and spices (Abera, 1995; Asfaw, 1997; Habtamu & Kelbessa, 1997). The crop has appreciable nutritional composition mainly of protein and calcium (Habtamu, Fekadu, & Gullelat, 2013; Habtamu & Kelbessa, 1997).

Anchote grows in wide environmental conditions from drier to cooler regions of Western and South Western region of Ethiopia (Endashaw, 2007). This makes the crop to be a potential food security crop. However, Anchote did not get adequate attention in terms of improving its productivity, and hence it has remained as one of underutilized crops in Ethiopia. So far, there has been little effort made to undertake varietal development to identify suitable cultivars with different desirable traits adaptable to the different agro‐ecological zone of Ethiopia, which makes its use to be limited to specific regions.

Research output on Anchote especially on its nutritional value is very limited and lack of scientific information on this crop is a common problem (Daba, Derebew, Wesene, & Waktole, 2012; Tilahun, Sentayehu, Amsalu, & Weyessa, 2014). The scanty information about the nutrition content including amino acid profile on the available Anchote accessions coupled with lack of awareness about the crop itself still makes it untapped. Information on the amino acid profile of the *C. abyssinica* accessions grown in Ethiopia is not available. Therefore, this study was conducted to evaluate the amino acid profiles and protein quality of tuber and leaf parts of five ex situ conserved accessions of Ethiopia.

## 2 Experimental

### 2.1 Sample preparation

Anchote tuber and leaf samples were harvested from Debre Zeit Agricultural Research Center experimental field from November 2011 to January 2012. Three healthy tubers from each accession were washed, peeled, and sliced using knife into small pieces and mixed thoroughly in order to prepare 400 g of samples which were placed in a paper bag and dried to a constant weight in a hot air oven (DHG‐ 9055A, Memment Germany) set at about 105°C. To prepare the leaf samples, 200 g of newly growing tips of leaves were cleaned and chopped into small pieces and oven dried at 70°C to a constant weight. The oven dried leaf and tuber samples were then milled to fine powder using an electrical miller (FW 100, Yusung Industrial Ltd, China). The powder was sieved using 0.425 mm mesh size. Finally, the dried powder samples were put into paper bags and packed with airtight polyethylene bags to store it in a refrigerator at 4°C until further analysis.

### 2.2 Crude protein determination

Crude protein content was estimated by the Kjeldhal method according to AOAC, (2000) using the official method 979.09. Accurately weighed 0.5 g sample was digested with a known quantity of concentrated H_2_SO_4_ (Sigma‐Aldrich, USA) in the Kjeltec digestion apparatus (Gerhardt vapodest, Germany). The digested material was distilled after the addition of alkali. The released ammonia was collected in 4% boric acid Kjeltec Automatic Distilling Unit. The resultant boric acid contained the ammonia released from the digested material, and then titrated with 0.1N hydrochloric acid (HCl) (Sigma‐Aldrich, USA). The protein content was determined by multiplying the nitrogen content by a factor of 6.25.

### 2.3 Amino acid analysis

Amino acid profile was determined according to Novus International inc. Amino Acid Assay for the determination of acid hydrolysable amino acids. The test was done using performic acid oxidation and acid hydrolysis of amino acids by Ninhydrin‐Derivatized analysis using amino acid analyzer (Hitachi L‐8800 Amino Acid Analyzer, Tokyo, Japan). The amino acids determined by this method were alanine (Ala), arginine (Arg), aspartic acid (Asp), cysteine (Cys), glutamic acid (Glu), glycine (Gly), histidine (His), isoleucine (Ile), leucine (Leu), lysine (Lys), methionine (Met), phenylalanine (Phe), proline (Pro), serine (Ser), threonine (Thr), tyrosine (Tyr), and valine (Val). Norvalene was used as an internal standard to normalize the recovery of each amino acid from injection to injection. The method was calibrated over the range of 0.08%–22.7% for each amino acid. Tryptophan (Trp) was not analyzed for the reason that acid hydrolysis results complete destruction of tryptophan and requires an alternative hydrolysis procedure for accurate quantification (Wathelet, 1999).

### 2.4 Evaluation of protein quality

Nutritional qualities of the protein in the leaf and tuber samples of Anchote were determined based on the obtained amino acid profiles. The parameters determined were as follows:

The proportion of total essential amino acids (TEAA) to the total amino acids (TAA) of the protein was calculated using the method of Chavan, McKenzie, and Shahidi (2001).

$$\begin{array}{cc} \text{TEAA/TAA}=\; & (\text{Ile + Leu + Lys + Met + Cys + Phe + Tyr + Thr + Trp + Val}\\ & +\;His)/(\text{Ala + Asp + Arg + Gly + Glu + His + Ile + Leu + Lys}\\ & \text{+}\;\;\text{Met + Cys}\text{+ Phe + Tyr + Pro + Ser + Thr + Trp + Val}) \end{array}$$

Amino acid score of the essential amino acid composition was calculated according to Chavan et al. (2001).

$$\begin{array}{cc} \text{Amino acid score} & =\text{(mg of amino acid per g test protein)}\;/\;\\ & \;\text{(mg of amino acid per g of FAO}\\ & \;/\text{WHO standard pattern)}\times 100 \end{array}$$

Essential amino acid index (EAAI) was calculated according to Ijarotimi and Keshinro (2011).

$$\mathrm{EAAI}=\sqrt{\left(n\&(100a\times 100b\times \ldots 100j)/(av\times bv\ldots jv)\right)}$$

Where:

*n*  =  number of essential amino acids, a, b …..j = represent the concentration of essential amino acids (histidine, isoleucine, leucine, lysine, methionine, phenylalanine, threonine, and valine,) in the tested sample and av, bv…..jv = content of the same amino acids in standard protein (%) (egg or casein), respectively.

Predicted biological value (P‐BV) was calculated according to Mune, Minka, Mbome, and Etoa (2011).

P−BV=1.09×EAAI−11.7

The predicted protein efficiency ratio (P‐PER) calculated by the regression equations as cited by Mune et al. (2011).

P−PER=−0.468+0.454(LEU)−0.105(TYR)

The nutritional index was calculated according to Ijarotimi and Keshinro (2013).

Nutritionalindex(%)=EAAI×%protein/100

## 3 Results and Discussion

### 3.1 Crude protein content

The crude protein content in Anchote tubers of the tested five accessions ranged from 10.70 ± 0.26% (“223090‐1”) to 13.72 ± 0.10% (“223097”) with a mean value of 12.06% (Table 1). No significant difference (*p* > .05) was observed between “223090‐1” (10.82%) and “NJ” (10.70%) accessions. However, there was a significant (*p* < .05) difference between the other three and these two accessions. The crude protein content of Anchote tuber in this study was stuck between the range value (4.6%–16.4%) reported by Desta (2011), but higher than the values (3.00%–3.20%) documented by others (EHNRI, 1997; Habtamu & Kelbessa, 1997; Habtamu et al., 2013). Our result is in close agreement with values reported for yam (*Dioscorea alata*) (10.27%), taro (*Colocasia Esculenta*) (11.00%), and wild yam (*Dioscorea oppositifolia var. dukhumensis)* (13.80%) (Arinathan, Mohan, & Maruthupandian, 2009; Ezeocha & Ojimelukwe, 2012; Melese & Negussie, 2015) In contrast protein content of Anchote tuber was superior than jicama (*Pachyrhizus erosus),* potato *(Solanum tuberosum), and* sweet potato *(Ipomoea batatas)* 1.23, 2.73, and 0.57% (Noman, Hoque, Haque, Pervin, & Karim, 2007), cassava (*Manihot esculenta*) 1.00 to 3.00% (Montagnac, Davis, & Tanumihardjo, 2009), “Amochi” (*Arisaema schimperianum*) 0.56%–0.86% (Andargachew, Admasu, Girma, Bjørnstad, & Appelgren, 2011), and yams (*Dioscorea spp*.) 1.00%–3.00% (Shewry, 2003).

**Table 1:** Proximate composition of Anchote tuber and leaf of five accessions

Accessions	Crude protein (%)
Tuber	
223097	13.72 ± 0.10^a^
223087‐1	13.25 ± 0.12^b^
223085	11.80 ± 0.15^c^
223090‐1	10.82 ± 0.27^de^
NJ	10.70 ± 0.26^de^
Leaf	
223109‐1	35.42 ± 0.05^a^
223090‐1	34.58 ± 0.29^a^
DIGGA‐1	34.00 ± 0.19^a^
KICHI	31.21 ± 0.28^b^
240407‐1	30.38 ± 0.01^bc^

Values are expressed as means ± standard deviations (SD); Means followed by different superscript letters in the same column are significantly different (*p* < .05).

The crude protein content in leaves was ranged between 30.38 ± 0.01% (“240407‐1”) and 35.42 ± 0.05% (“223109‐1”) with mean crude protein content of 33.12% (Table 1). No significant difference (*p* > .05) was observed in crude protein content of the top three accessions: “223109‐1” (35.42%), “223090‐1” (34.58%), and “DIGGA‐1” (34.00%). However, the observed variation in the crude protein content of these three accessions and the rest of the accessions was significant (*p* < .05). However, the crude protein content recorded for Anchote leaves was higher than the value reported for sweet potato leaves (24.85%) (Antia, Akpan, Okon, & Umoren, 2006). The mean protein content of Anchote leaves in this study was much higher than *Xanthosoma sagittifolia* (4.65 + 0.02%), *Amaranth cruentus* (4.46 + 0.03%), *Talinum triangulare* (5.10 + 0.01%), and *Moringa oleifera* (6.60 + 0.02%) (Kwenin, Wolli, & Dzomeku, 2011). However, it was lower than *Moringa oleifera* leaf at different maturity stages, that is, 10th (early stage), 15th (Mid stage), and 20th (late stage) week after pruning (23.7 ± 0.12–28.08 ± 2.75%) (Bamishaiye, Olayemi, Awagu, & Bamshaiye, 2011). Lower crude protein contents were reported for fresh leaves of pumpkin (4.58%), onion (5.30%) (Pedavaoh & Kavaarpuo, 2014), *Amaranthus aquatica* (3.50%), *Telfaira occidentalis* (4.70%) (Gladys, 2011), kale (*Brassica oleraceae*) (11.67%) (Emebu & Anyika, 2011), and raw *Amaranthus hybridus* (4.3%) (Mepba, Eboh, & Banigo, 2007) compared to the present crude protein contents for Anchote leaves (33.12%). However, the mean crude protein content recorded for Anchote leaves (33.12%) is comparable to the value reported for sweet potato leaves (24.85%) (Antia et al., 2006). This result tends to suggest that Anchote leaves have higher protein content than tubers. Therefore, leaves of Anchote can be good source of protein with the evidence that confirms any plant foods which have the potential to provide about 12.00% of their calorific value from protein are considered good source of protein (Aberoumand, 2010; Effiong, Ibia, & Udofia, 2009; Nwofia, Victoria, & Blessing, 2012).

### 3.2 Amino acid composition

Proteins are composed of different amino acids and hence the nutritional quality of a protein determined by the content, proportion, and availability of its amino acids (Becker, 2007). The result for amino acid profile of five Anchote accessions selected based on their protein content is presented in Table 2.

**Table 2:** Amino acid composition in selected five accessions of Anchote tuber and leaf powder (g/100 g protein dry weight basis)

Amino acids	Tuber					Leaf				
	Accessions									
	223097	223087‐1	223085	223090‐1	NJ	223109‐1	223090‐1	DIGGA‐1	KICHI	240407‐1
Essential amino acids										
His	0.62	0.56	0.63	0.89	0.70	1.34	1.44	1.36	2.39	1.62
Ile	2.73	2.48	2.71	3.45	4.31	3.23	3.39	3.24	5.06	3.56
Leu	3.59	3.12	3.43	4.34	5.32	5.21	5.49	5.15	5.42	5.65
Lys	2.42	1.92	2.35	3.05	3.61	3.61	3.74	3.57	4.07	4.01
Met	0.31	0.40	0.36	0.30	1.10	0.89	0.94	0.87	0.98	0.96
Phe	1.72	1.52	1.62	2.17	2.61	2.96	2.85	2.87	3.42	3.39
Thr	2.81	2.40	2.71	3.35	4.01	3.26	3.39	3.21	3.82	3.63
Val	2.89	2.48	2.98	3.55	4.51	4.03	4.25	4.02	4.12	4.42
TEAA	17.10	14.88	16.78	21.09	26.18	24.53	25.50	24.29	29.28	27.25
Conditionally essential amino acids										
Arg	8.51	9.52	8.03	6.50	7.02	3.12	3.43	5.07	6.28	3.75
Cys	1.41	1.36	1.62	1.68	1.81	3.55	3.29	3.64	2.92	2.97
Gly	2.73	2.56	2.80	3.25	4.51	5.84	6.18	5.35	4.69	6.39
Pro	0.94	0.80	0.90	0.69	0.60	1.78	2.01	2.66	2.30	2.05
Tyr	0.86	0.72	0.90	1.08	1.50	1.49	1.56	2.18	1.27	1.67
TCEA	14.44	14.96	14.26	13.20	15.45	15.78	16.47	18.90	17.46	16.83
Nonessential amino acids										
Ala	3.43	3.12	3.43	3.94	5.92	5.58	5.88	5.03	5.17	6.22
Asp	4.76	4.08	4.60	5.71	7.42	8.39	9.21	7.49	9.35	10.28
Glu	5.62	5.36	5.23	3.84	3.31	8.86	9.21	7.87	8.17	10.47
Serine	3.20	2.72	3.16	3.74	4.61	4.17	4.33	4.32	4.47	4.64
TNEA	17.02	15.28	16.42	17.24	21.26	27.00	28.63	24.71	27.14	31.61
TAA	48.55	45.12	47.46	51.53	62.89	67.31	70.60	67.89	73.89	75.69

TEAA, Total essential amino acid; TCEA, Total conditionally essential amino acid; TNEAA, Total nonessential amino acid; TAA, Total amino acid.

The amino acids profile of Anchote tuber showed that Arg (6.50–9.52 g/100 g protein) was the highest, while Met (0.30–0.40 g/100 g protein) was the least in concentration for four accessions, “223097”, “223087‐1”, “223085”, and “223090‐1”. Whereas, in accession “NJ” Asp (7.42 g/100 g protein) was the highest and Pro was the least (0.60 g/100 g protein) in concentration. In Anchote leaf, Glu (7.87–10.47 g/100 g protein) scored the highest value except in accession “KICHI” where Asp (9.35 g/100 g protein) was the most abundant, whereas Met was the limiting amino acid in all accessions. Similar to the present finding high amount of Glu was observed in previous reports on plant‐based protein (Adeyeye, 2004; Ijarotimi & Keshinro, 2013; Olaofe, Adeyemi, & Adediran, 1994). The most abundantly found amino acids in Anchote tuber (Arg and Asp) were in agreement with the reported values for *Dioscorea* species and cassava tubers (Babu, Nambisan, Sundaresan, & Abraham, 2007; Montagnac et al., 2009). As of Anchote leaf the amino acids with highest concentration (Glu and Asp) were in accordance with the reported values for *Amaranths hybridus* leaves (Akubugwo, Obasi, Chinyere, & Ugbogu, 2007).

The essential amino acids (His, Ile, Leu, Lys, Met, Phe, Thr, Try, and Val) for Anchote accessions ranged from 32.98% to 41.63% (mean = 37.22%) in tuber and from 35.78% to 39.63% (mean = 36.79%) in leaf part. Conditionally essential amino acids (Arg, Cys, Gly, Pro, and Tyr) of the tuber ranged from 24.56% to 33.16% (mean = 28.62%), whereas the leaf was from 22.24% to 27.83% (mean = 24.10%). Nonessential amino acids (Ala, Asp, Glu, and Ser) were between 33.87 – 42.92% (mean = 34.16%) for tuber and 36.39%–41.76% (mean = 39.11%) for leaf of Anchote.

Leu was the dominant essential amino acid in all Anchote accessions ranged from 3.12 to 5.32 g/100 g protein for tuber and from 5.15 to 5.65 g/100 g protein for leaf. Accession “NJ” in tuber and “240407‐1” in leaf were recorded the highest Leu content. Met was the least in concentration among all essential amino acids in both tuber and leaf part, which was in agreement with germplasm accessions of *Dioscorea* species (Babu et al., 2007) and sweet potato cultivars (Van Hal, 2000). Arg was the most abundant amino acid among conditionally essential amino acids of all accessions in tuber part and in one of the accession evaluated for leaf part (“KICHI”) with values ranging from 6.28 to 9.52 g/100 g protein. Gly was the highest amino acid in leaf of Anchote for the rest of accessions. With regard to nonessential amino acids Glu was dominantly found in tuber (5.23–5. 62 g/100 g protein) and leaf (7.87–10.47 g/100 g protein) with the exception of accession “223090‐1” and “NJ” in tuber, and “KICHI” in leaf revealed Asp the highest of all nonessential amino acid. These results are comparable with most vegetable protein (El‐Adawy, Rahma, El‐Bedawey, & Gafar, 2001; Mune et al., 2011; Ogunlade, Olaifa, Adeniran, & Ogunlade, 2011; Sánchez‐Vioque, Clemente, Vioque, Bautista, & Millán, 1999). The average percentage of nonessential amino acids was higher in concentration (62.78% and 63.21%) than essential amino acids (37.22% and 36.79%) in both tuber and leaf part, respectively. Similar observations were reported in previous studies (Akubugwo et al., 2007; Aremu, Olaofe, & Akintayo, 2006; Hassan & Umar, 2006).

The total amino acid (TAA) content of Anchote ranged from 45.12 to 62.89 g/100 g protein in tuber and from 67.31 to 75.69 g/100 g protein in leaf. The amino acid content was higher in leaf (71.08 g/100 g protein) compared to the tuber (51.11 g/100 g protein). This could relate to the highest crude protein content that was recorded for leaf (35.42%) compared to the tuber (13.72%). This observation is agreed with the report that states leaves and vines of sweet potato were high in total amino acids than the tubers (Kenyon, Anandajayasekeram, Ochieng, & Ave, 2006).

A balanced or high‐quality protein contains essential amino acids in ratios commensurate with human needs. This can be determined by comparing the amino acid contents of various proteins with the FAO reference pattern. The FAO reference pattern based on the essential amino acid requirements of young children (1–2 years) is considered the preferred reference protein (Cheftel, Cuq, & Lorient, 1985). Thus, the average proportions of the essential amino acid profile of Anchote tuber and leaf were compared with the (WHO, 2007) reference pattern for the preferred age group as shown in Table 3.

**Table 3:** Comparison of mean (*n* = 5) essential amino acid composition (g/100 g protein) of Anchote tuber and leaf with the WHO standard reference pattern

EAAs	Tuber	Leaf	WHO* reference pattern (1–2 years age children)
Histidine	0.68	1.63	1.80
Isoleucine	3.14	3.70	3.10
Leucine	3.96	5.38	6.30
Lysine	2.67	3.80	5.20
Methionine	0.49	0.93	–
Phenylalanine	1.93	3.10	–
Threonine	3.06	3.46	2.70
Tryptophan	–	–	0.74
Valine	3.28	4.17	4.20
SAAs	2.07	4.20	2.60
AAAs	2.94	4.73	4.60

Source: *WHO (2007), Essential amino acids (EAAs), Sulfur amino acids (SAAs), Aromatic amino acids (AAAs).

All the essential amino acids were found in both tuber and leaf of Anchote except tryptophan (Trp), which was not determined in this study. Met and His were found in limited amount for tuber and leaf part, and this limitation might be explained by two possible reasons; they might be denaturized during analysis or their values are very limited in Anchote. The low availability of Met is in accordance with the previous studies (Montagnac et al., 2009; Van Hal, 2000). To compensate this limitation in Anchote, additional consumption of animal or plant proteins such as milk, egg, lentils, and pulses are highly recommended (Andini, Yoshida, & Ohsawa, 2013).

Essential amino acids Ile, 3.70; Thr, 3.46; sulfur containing amino acids (SAAs) 4.20; and Aromatic amino acids (AAAs), 4.73 g/100 g protein in leaf, and Ile,3.14 and Thr, 3.06 g/100 g protein in tuber of Anchote were higher than the reference standards (WHO, 2007) (Ile 3.10;Thr, 2.70; SAAs, 2.60 and AAAs, 4.60 g/100 g protein). These results suggests that Anchote can be exploited for those essential amino acids which are found in adequate amount in either of its edible part to enhance protein quality especially when preparing weaning/complimentary food products.

### 3.3 Protein quality

The nutritional quality of a food protein depends on the kinds and amounts of amino acids it contains, and represents a measure of the efficiency with which the body can utilize the protein (Chawanje, Barbeau, & Grün, 2001). The protein quality of Anchote tuber and leaf were determined based on their amino acid profile and presented in Table 4. In Anchote leaf, the content of SAAs (Met + Cys) was 4.20 g/100 g protein and in its tuber, it was 2.07 g/100 g protein. The leaf SAAs (4.20 g/100 g protein) was relatively higher than the required reference pattern (2.2–2.8 g/100 g protein or 22–28 mg/g protein) set by WHO, (2007) for different age group although the tuber sample was below the recommended value. This might be due to Anchote leaf protein contains substantially more Cys than Met which is in close agreement with many vegetable proteins, especially the legumes (WHO, 2007). The AAAs (Phe + Tyr) of Anchote tuber and leaf were 2.94 and 4.73 g/100 g protein, respectively. The content of AAAs of Anchote leaf were within the ideal range (3.8–4.6 g/100 g protein or 38–46 mg/g protein) of amino acids requirement suggested by WHO, (2007) for different age groups except for ideal infant (5.2 g/100 g protein or 52 mg/g protein) requirement.

**Table 4:** Estimated nutritional quality of protein for Anchote tuber and leaf samples based on their amino acid profile

Nutritional quality of amino acids	Tuber	Leaf
TSAA(Meth+Cys) (g/100 g protein)	2.07	4.20
TArAA (Phe+Tyr) (g/100 g protein)	2.94	4.73
Leu/Ileu ratio	1.26	1.46
TEAA/TAA%	37.57	36.82
TNEAA/TAA%	62.43	63.18
TEAA/TNEAA ratio	0.60	0.58
P‐PER	1.22	1.80
EAAI (%)	35.28	53.93
P‐BV (%)	26.76	47.09
Nutritional index (%)	4.11	17.71
Amino acid score	73	108

TArAA, Total aromatic amino acids; TSAA, Total sulfur amino acids; TEAA, Total essential amino acids; TNEAA, Total nonessential amino acids; TAA, Total amino acids; His, Histidine; Arg, Arginine; Leu, Leucine; Ile, Isoleucyne; PER, Protein efficiency ratio; EAAI, Essential amino acid index, BV, Biological value.

The Leu/Ile ratio of Anchote tuber (1.26) and leaf (1.46) were lower than the flour (2.10) and protein concentrate (2.21) of Bambara bean (Mune et al., 2011). According to Deosthale, Mohan, & Rao, (1970) excess Leu content in foods interferes with the utilization of Ile and Lys. The percentage of essential to total amino acids (TEAA/TAA) was 37.57% for tuber and 36.82% for leaf of Anchote. The average predicted protein efficiency ratios (P‐PER) for tuber was 1.22 and for leaf, it was 1.80. This P‐PER value was higher than sorghum ogi (0.27) (Oyarekua & Eleyinmi, 2004) and *L. sativum* (negative to 0.03) (Salunkhe & Kadam, 1989), but lower than whole hen's egg (2.88) (Paul, Southgate, & Russell, 1980), reference casein (2.50) and modified corn ogi (4.06) (Oyarekua & Eleyinmi, 2004). However, our results were favorably comparable to cowpea (1.21), pigeon pea (1.82), and millet ogi (1.62) (Oyarekua & Eleyinmi, 2004; Salunkhe & Kadam, 1989). The essential amino acid index (EAAI) of Anchote tuber (35.28%) were higher than fermented popcorn‐African locust bean (29.19%) and lower than fermented popcorn‐bambara groundnut (40.72%) and fermented popcorn‐African locust bean‐bambara groundnut (47.38%), whereas Anchote leaf (53.93%) was higher than the EAAI in the blended flour samples (Ijarotimi & Keshinro, 2013). According to Ijarotimi & Keshinro, (2011), EAAI can be used as a rapid tool to evaluate the protein quality of food formulations.

The Predicted biological value (P‐BV) of Anchote tuber sample (26.76%) was lower than Anchote leaf sample (47.09%). The P‐BV of Anchote tuber has higher value compared to fermented popcorn‐African locust bean flour blend (20.13%), *Citrullus colocynthis* (12.83%), fermented popcorn (3.15%), and germinated popcorn (10.53%) (Ijarotimi & Keshinro, 2011, 2013; Ogundele, Oshodi, & Amoo, 2012). Whereas, the P‐BV of the leaf was higher than that of beach pea protein isolates (36.5%–40.13%), raw popcorn flour (36.45%), flour blends made from fermented popcorn‐bambara groundnut (32.69%) and fermented popcorn‐African locust bean‐bambara groundnut (39.94%) (Chavan et al., 2001; Ijarotimi & Keshinro, 2011, 2013). The P‐BV obtained from Anchote leaf was in agreement with the suggested biological value (45%) for plant‐based proteins (Ogundele et al., 2012). The nutritional index for Anchote tuber was 4.11%, whereas for the leaf part it was 17.71%. Anchote leaf nutritional index was higher than formulated complementary food (5.98%–12.73%) of plant‐based protein (Ijarotimi & Keshinro, 2013). The amino acid score is the ratio of the amino acid content in the sample protein to the content of the same amino acid in the requirement pattern. The amino acid score of Anchote tuber (73) was lower when compared to beach pea protein isolates (108–110), whereas the content in Anchote leaf (108) had a similarity with this report (Chavan et al., 2001).

## 4 Conclusion

The study investigated the protein content, amino acid profile, and nutritional quality of leaf and tuber samples from different Anchote accessions. The leaf sample was ranked best compared to the tuber sample in crude protein and amino acid content as well as protein quality. Anchote can be exploited for those essential amino acids (Leu, Ile, Thr, SAAs, and AAAs) which are found in adequate amount in either of its edible part to enhance protein quality especially when preparing plant‐based weaning/complimentary food products. The dominant essential amino acid was Leu in all Anchote accessions and accession “NJ” in tuber and accession “240407‐1” in leaf was recorded the highest Leu content. Met and His were found in limited amount in both tuber and leaf part. The amino acid composition also varies among accessions in both tuber and leaf samples. Therefore, through selection and hybridization of protein‐rich accessions it can be possible to overcome low level of protein. Moreover, genetic modification can be applied to improve the availability and quality of protein.

## Conflict of Interest

None declared.
